# Structured diabetes care routines in cardiac rehabilitation are associated with increased diabetes detection and improved treatment after myocardial infarction: a nationwide observational study

**DOI:** 10.1186/s12933-024-02425-6

**Published:** 2024-09-03

**Authors:** Bashaaer Sharad, Nils Eckerdal, Martin Magnusson, Halldora Ögmundsdottir Michelsen, Amra Jujic, Matthias Lidin, Linda Mellbin, Nael Shaat, Ronnie Pingel, John Wallert, Emil Hagström, Margrét Leósdóttir

**Affiliations:** 1 Department of Clinical Sciences Malmö, Lund University, Jan Waldenströms gata 15 plan 3, 205 02 Malmö, Sweden; 2 Department of Cardiology, Skane University Hospital, Malmö, Sweden; 3 Department of Statistics, Uppsala University, Uppsala, Sweden; 4 Department of Emergency Medicine and Geriatrics, Helsingborg Hospital, Helsingborg, Sweden; 5 EXODIAB: Excellence of Diabetes Research in Sweden, Lund University, Malmö, Sweden; 6 Department of Medicine Solna, Karolinska Institute, Stockholm, Sweden; 7 Department of Cardiology, Karolinska University Hospital, Stockholm, Sweden; 8 Department of Endocrinology, Skane University Hospital, Malmö, Sweden; 9 Department of Clinical Neuroscience, Center for Psychiatry Research, Karolinska Institutet, Stockholm Health Care Services, Region Stockholm, Sweden; 10 Department of Medical Sciences, Cardiology, Uppsala University, Uppsala, Sweden; 11 Wallenberg Center for Molecular Medicine, Lund University, Lund, Sweden; 12 Hypertension in Africa Research Team, North-West University, Potchefstroom, South Africa

**Keywords:** Cardiac rehabilitation, Myocardial infarction, Diabetes, Secondary prevention

## Abstract

**Background:**

Despite the detrimental impact of abnormal glucose metabolism on cardiovascular prognosis after myocardial infarction (MI), diabetes is both underdiagnosed and undertreated. We investigated associations between structured diabetes care routines in cardiac rehabilitation (CR) and detection and treatment of diabetes at one-year post-MI.

**Methods:**

Center-level data was derived from the Perfect-CR survey, which evaluated work routines applied at Swedish CR centers (*n* = 76). Work routines involving diabetes care included: (1) routine assessment of fasting glucose and/or HbA1c, (2) routine use of oral glucose tolerance test (OGTT), (3) having regular case rounds with diabetologists, and (4) whether glucose-lowering medication was adjusted by CR physicians. Patient-level data was obtained from the national MI registry SWEDEHEART (*n* = 7601, 76% male, mean age 62.6 years) and included all post-MI patients irrespective of diabetes diagnosis. Using mixed-effects regression we estimated differences between patients exposed versus. not exposed to the four above-mentioned diabetes care routines. Outcomes were newly detected diabetes and the proportion of patients receiving oral glucose-lowering medication at one-year post-MI.

**Results:**

Routine assessment of fasting glucose/HbA1c was performed at 63.2% (*n* = 48) of the centers, while 38.2% (*n* = 29) reported using OGTT for detecting glucose abnormalities. Glucose-lowering medication adjusted by CR physicians (*n* = 13, 17.1%) or regular case rounds with diabetologists (*n* = 7, 9.2%) were less frequently reported. In total, 4.0% of all patients (*n* = 304) were diagnosed with diabetes during follow-up and 17.9% (*n* = 1361) were on oral glucose-lowering treatment one-year post-MI. Routine use of OGTT was associated with a higher rate of newly detected diabetes at one-year (risk ratio [95% confidence interval]: 1.62 [1.26, 1.98], *p* = 0.0007). At one-year a higher proportion of patients were receiving oral glucose-lowering medication at centers using OGTT (1.22 [1.07, 1.37], *p* = 0.0046) and where such medication was adjusted by CR physicians (1.31 [1.06, 1.56], *p* = 0.0155). Compared to having none of the structured diabetes care routines, the more routines implemented the higher the rate of newly detected diabetes (from 0 routines: 2.7% to 4 routines: 6.3%; p for trend = 0.0014).

**Conclusions:**

Having structured routines for diabetes care implemented within CR can improve detection and treatment of diabetes post-MI. A cluster-randomized trial is warranted to ascertain causality.

**Supplementary Information:**

The online version contains supplementary material available at 10.1186/s12933-024-02425-6.

## Background

Abnormal glucose metabolism is common in patients with ischemic heart disease and is associated with a considerably higher risk of mortality and recurrent cardiovascular events [1]. The reported prevalence of diabetes mellitus in patients with myocardial infarction (MI) is 20–25% [2–4]. Further two-thirds of patients with MI without known diabetes have undiagnosed diabetes or pre-diabetes (impaired fasting glucose or impaired glucose tolerance) when tested via fasting glucose, hemoglobin A1c (HbA1c) or an oral glucose tolerance test (OGTT) [5]. Both patients with diabetes and those with pre-diabetes have a worse prognosis than patients with normal glucose metabolism [6, 7].

Despite improvements in diagnostic methods, a considerable proportion of MI patients with diabetes and pre-diabetes remain undiagnosed and therefore also untreated [5, 8]. In recent years, new treatments (e.g., glucagon-like peptide-1 receptor agonists [GLP-1RA] and sodium-glucose cotransporter-2 [SGLT-2] inhibitors provide additional prognostic benefit beyond optimal metabolic and risk factor control [9]. Given this background, there is a need to improve screening for, and treatment of, abnormal glucose metabolism in patients with MI to improve long-term prognosis.

According to international guidelines on diabetes and cardiovascular disease, fasting glucose and HbA1c should be routinely measured in all patients with MI to confirm or exclude a diabetes diagnosis [5, 10]. An OGTT may be considered in cases where a diabetes diagnosis is still unclear [5]. If abnormal glucose metabolism is identified, management of risk factors including lifestyle modification, adequate secondary preventive treatment and glycemic control should be applied to decrease the risk for recurrent cardiovascular events [5, 10].

Despite guideline recommendations, follow-up at many cardiac rehabilitation (CR) centers for post-MI patients does not routinely include assessment of glucose metabolism [2–4]. Also, pharmaceutical treatment and lifestyle modifications in patients with concomitant MI and diabetes are insufficient [11]. In Sweden, CR for post-MI patients is well integrated into routine healthcare and holds a high international standard [4, 12–14]. However, there is considerable variation in both target attainment and the work routines applied at Swedish CR centers to reach treatment targets for risk factors [4, 12]. It is unknown to which extent structured routines for diabetes care are integrated into CR in Sweden, and whether having such routines implemented within CR improves detection and treatment of glucometabolic abnormalities for post-MI patients.

The main purpose of this study was to (1) describe how screening and management of glucometabolic abnormalities is organized at CR centers in Sweden and (2) explore associations between work routines for management of glucometabolic abnormalities at center-level and rate of newly detected diabetes and treatment at patient-level at one-year post-MI.

## Methods

### Study design

This was an observational survey- and registry-based cohort study.

### Data collection

Three databases were used in this study: the Perfect-CR study, the national MI registry SWEDEHEART [4] and The Statistics Sweden registry [15].

The Perfect-CR study was a survey-based study which evaluated the work routines followed at CR centers in Sweden in 2016 [12]. All 78 centers actively following post-MI patients in Sweden at the time completed the survey and missing data was minimal (< 3%). Details of the study procedure have previously been published [12]. In the current study we focused on the part of the survey exploring work routines pertaining to diabetes management during CR. These included four work routines, defined as the study exposures: (1) routine assessment of fasting glucose and/or HbA1c values by a nurse at the start of the CR program (laboratory measures performed at index MI hospitalization or prior to CR start), (2) routine use of OGTT during follow-up, (3) having regular joint case rounds with diabetologists, and (4) initiation and/or adjustment of glucose-lowering medication by the CR center’s physicians. The use of OGTT could be performed in all patients without known diabetes or on a selection of patients (high normal HbA1c/fasting glucose). Out of the 78 CR centers participating in Perfect-CR, two centers had no follow-up data and were excluded from further analysis.

Patient baseline and outcome data were extracted from the SWEDEHEART and Statistics Sweden registries. SWEDEHEART is a nationwide registry for cardiac disease. The registry includes patient data from all hospitals in Sweden that treat and follow up patients with acute MI. Data on acute care is collected at coronary care units and follow-up data is collected at CR centers at 2-months and one-year post-MI [16, 17]. All coronary care units and CR centers in the country report to SWEDEHEART. The registry data includes information on comorbidities, previous medical history and medications, laboratory analyses, risk factors, psychosocial- and lifestyle variables, and readmissions [4]. Patient inclusion is shown in Figure S1. Inclusion criteria were: 1) discharged alive after hospitalization for type-1 MI, 2) age 18–74 years, and 3) attending CR in the year preceding administration of the Perfect-CR survey. The timeframe was chosen to match the time in which patients attended CR with the time reflected in the Perfect-CR survey answers. There were no exclusion criteria. Diabetes (type 1 or 2) in SWEDEHEART is defined as a diagnosis in medical records and/or by the patient being prescribed glucose-lowering medication. A diagnosis of pre-diabetes is not registered in SWEDEHEART. No documentation of how a diagnosis has been made is available in SWEDEHEART and can vary between centers. At the time of the study, the type of diabetes was not registered in SWEDEHEART. Hence, in this study a distinction between different types of diabetes was not possible. Also, variables reflecting diabetes treatment in SWEDEHEART at the time were “Treatment with insulin (yes/no)” and “Treatment with other oral glucose-lowering medications (yes/no)”. Further classification of glucose-lowering medication was not registered and injection therapy other than insulin was uncommon [18].

Statistics Sweden is the Swedish government agency responsible for producing official statistics including data from Swedish health care units [15]. Data from Statistics Sweden used in this study included baseline data on socioeconomic status including country of birth, disposable household income, marital status, occupational status, and level of education [15].

Center-level data were merged with patient-level data by linking each patient to the specific CR center where the patient underwent their follow-up [19]. The final dataset used in the current study included all patients (*n* = 7601) discharged with an MI diagnosis (ICD-10 codes I21) between Nov 2015 and Oct 2016 and subsequently followed at the 76 included CR centers.

### Outcome definition

Outcomes were (1) rate of newly detected diabetes cases and (2) the proportion of patients receiving glucose lowering treatment other than insulin at a one-year follow-up visit registered in SWEDEHEART. If one-year data was missing, data from the 2-month follow-up visit was used. Pre-diabetes was not included in the newly detected diabetes outcome as information on pre-diabetes diagnosis is not available in SWEDEHEART. Also, as it is uncommon for CR physicians to initiate or adjust insulin, we only included treatment with oral glucose-lowering medication in the treatment outcome. All patients irrespective of diabetes diagnosis were included in the diabetes treatment outcome analysis to ascertain inclusion of patients with pre-diabetes, that might be treated with oral glucose-lowering medication, predominantly metformin [20].

### Statistical analysis

Baseline data are reported as frequencies and percentages (%) medians and interquartile ranges (Q1, Q3), or means and standard deviations (SD) as appropriate. Exposures were modelled in three different ways: (i) having two to four routines implemented defined as exposure versus having zero or one routine implemented defined as reference; (ii) each working routine defined as a separate exposure with not having that routine implemented defined as the reference; and (iii) the number of implemented routines from zero to four, with zero being the reference. The association between the exposures and the outcomes were estimated as incidence changes (risk differences) and incidence ratios (risk ratios). Both measures were based on a mixed-effects linear model, with random intercepts for each CR center. The risk ratio was estimated as the sum of the crude control-group incidence and the risk difference, divided by the control-group incidence. Our linear mixed model did not include crude incidence and did thus not calculate in the RR variance. Instead, the variance of the risk ratio estimate was calculated using the delta method, assuming independence of the risk difference and the control-group crude incidence. Covariates comprised (a) patient-related variables including age, gender, comorbidities, body max index, smoking status, marital status, education attainment, disposable income, and participation in educational and training programs as part of the comprehensive CR program; and (b) center-related variables including composition of the CR team, qualifications of CR team members, team spirit, audit data being used for quality control, regular team meetings, content of the comprehensive CR program, size of the CR center and whether it was situated at a university hospital. All covariates are presented in a Directed Acyclic Graph (DAG) in Figure S2.

To maximize statistical power and avoid biasing of estimates from using only complete cases, multiple imputation was implemented. With the overall rate of missingness among covariates being only 1.3%, simple hot deck imputation was used. Missing values on individual-level covariates were replaced by the value of a randomly selected individual from the same CR center. Missing values on CR center-level covariates were replaced by the value of a randomly selected CR center. Thirty imputed data sets were created, and the results were combined using Rubin’s rules.

 [21]. Statistical significance was set to *p* < 0.05. Data was analyzed using IBM SPSS (version 27, Chicago, Illinois, USA) and R version 4.2.2. According to relevant literature, 20 data sets would be suitable, yet 30 were used to account for differences between our data and simulations [22].

## Results

### Center-level baseline characteristics

Out of the 76 CR centers participating in Perfect-CR where follow-up data on patient-level was available, nine centers (11.8%) were based at university hospitals. The median number of patients eligible for follow-up per center in 2016 was 101 (68, 164). Fasting glucose and/or HbA1c were routinely evaluated at CR program initiation at 63.2% (*n* = 48) of the CR centers, while 38.2% (*n* = 29) of the centers reported routinely performing OGTT. At 17.1% (*n* = 13) of the CR centers the physicians independently initiated and/or adjusted glucose-lowering medication and 9.2% (*n* = 7) reported having regular joint case rounds with diabetologists. Most centers had none (23.7%, *n* = 18) or one (36.8%, *n* = 28) of the work routines for structured diabetes care in place, while 39.4% (*n* = 30) of the CR centers reported having two or more routines implemented. Only 2.6% (*n* = 2) of the centers reported having all four work routines implemented. Further details can be seen in Table 1.

**Table 1:** Diabetes care work routines applied at cardiac rehabilitation centers in Sweden as surveyed in the Perfect-CR survey

Questionnaire item	*N* (%)
CR centers	76
Fasting glucose and/or HbA1c levels are routinely evaluated at the start of the CR program	48 (63.2)
Use of OGTT*	
OGTT is performed on all patients	7 (9.2)
OGTT is performed on a selection of patients	22 (28.9)
We do not perform OGTT on our patients post-MI	46 (60.5)
Unknown	1 (1.3)
Glucose-lowering medication is initiated and/or adjusted by the CR center physicians	13 (17.1)
Regular joint case rounds with diabetologists at the CR center	7 (9.2)

*In patients without previous diabetes diagnosis. HbA1c: hemoglobin A1c; CR: cardiac rehabilitation; OGTT: oral glucose tolerance test; MI: myocardial infarction

### Patient-level baseline characteristics

In total, 7601 patients followed at the 76 CR centers were included. Mean age of the patients was 62.6 *±* 8.7 years and 76% were male. The proportion of patients with previously known or newly detected diabetes during hospitalization was 22.5% (*n* = 1710), out of which 80.8% were treated with any glucose-lowering agent (oral or injection) at discharge. Diabetes prevalence at one-year follow-up was 26.5% (*n* = 2013) with 86.3% of the patients being treated with any glucose-lowering agent. Further patient-level baseline characteristics are shown in Table 2.

**Table 2:** Baseline characteristics at patient-level

	Total sample	0–1 routines followed	2–4 routines followed	*P*-value	Missing, *n* (%)
	7601 (100.0)	4947 (65.0)	2654 (35.0)		
**Demographics**					
Male, n (%)	5744 (76.0)	3770 (76.2)	2004 (75.5)	0.50	0 (0)
Age, years	62.6 *±* 8.7	62.7 + 8.7	62.5 + 8.6	0.86	0 (0)
**Risk factors and previous disease**					
Prevalent diabetes, n (%)	1522 (20.0)	1005 (20.3)	517 (19.5)	0.09	18 (0.2)
Active smoker, n (%)	2119 (27.9)	1331 (27.0)	788 (30.0)	0.003	186 (2.4)
Chronic heart failure, n (%)	364 (4.8)	217 (4.3)	147 (5.5)	< 0.001	184 (2.4)
Hypertension, n (%)	3576 (47.0)	2340 (47.3)	1236 (47.0)	0.80	34 (0.4)
Prior myocardial infarction, n (%)	1426 (18.8)	876 (17.7)	550 (20.7)	0.006	27 (0.4)
**Physiological and laboratory measures at baseline**					
Fasting plasma glucose, mmol/L	8.0 (3.5)	8.0 (3.5)	8.1 *±* 3.5	0.16	724 (9.5)
HbA1c, mmol/mol	44.0 (14.2)	43.9 (14.4)	44.1 *±* 13.9	0.50	5406 (71.1)
**Type of myocardial infarction**					
STEMI, n (%)	2966 (39.0)	1093 (22.1)	1063 (40.0)	0.18	0 (0)
NSTEMI, n (%)	4635 (61.0)	3044 (61.5)	1591 (60.0)	0.19	0 (0)
**Glucose-lowering treatment on hospital admission**					
Treatment with insulin, n (%)	681 (9.0)	457 (9.2)	224 (8.4)	< 0.001	118 (1.5)
Treatment with oral glucose lowering medication, n (%)	974 (12.8)	646 (13.1)	328 (12.4)	< 0.001	124 (1.6)
**Glucose-lowering treatment at hospital discharge**					
Treatment with insulin, n (%)	722 (9.5)	490 (10.0)	232 (8.7)	0.10	1 (0)
Treatment with oral glucose-lowering medication, n (%)	1080 (14.2)	686 (13.9)	394 (14.8)	0.24	0 (0)

Data are presented as count (%) or decimal mean (SD). HbA1c: hemoglobin A1c; DM: diabetes mellitus; STEMI: ST elevation myocardial infarction; NSTEMI: non-ST elevation myocardial infarction

### Analysis of routines

Routinely performed OGTT was the work routine which implementation differed the most between the dichotomized groups 0–1 routines (11%) vs. 2–4 routines (87%) (Table 3). Also, none of the CR centers where 0–1 routines were followed reported CR physicians to initiate and/or adjust glucose-lowering medication. Routine composition for individual routine exposures, by the number of patients per routine and the percentage of patients exposed/not exposed to other routines are shown in Table S1.

**Table 3:** The number and percentage of patients followed at CR centers applying the surveyed diabetes care work routines, by advancing number of routines (0–4 routines) and dichotomized (0–1 vs. 2–4 routines)

Number of routines	*n*	FG/HbA1c measured	OGTT routinely performed	Joint case rounds	Glucose lowering medication adjusted
0	2024	0 (0)	0 (0)	0 (0)	0 (0)
1	2919	2161 (74)	529 (18)	229 (8)	0 (0)
2	1890	1686 (89)	1546 (82)	256 (14)	292 (15)
3	478	478 (100)	478 (100)	170 (36)	308 (64)
4	284	284 (100)	284 (100)	284 (100)	284 (100)
0–1	4943	2161 (44)	529 (11)	229 (5)	0 (0)
2–4	2652	2448 (92)	2308 (87)	710 (27)	884 (33)

Data are presented as counts (%). FG: fasting glucose; HbA1c: hemoglobin A1c; OGTT: oral glucose tolerance test

### Outcome analyses

**Fig. 1:**
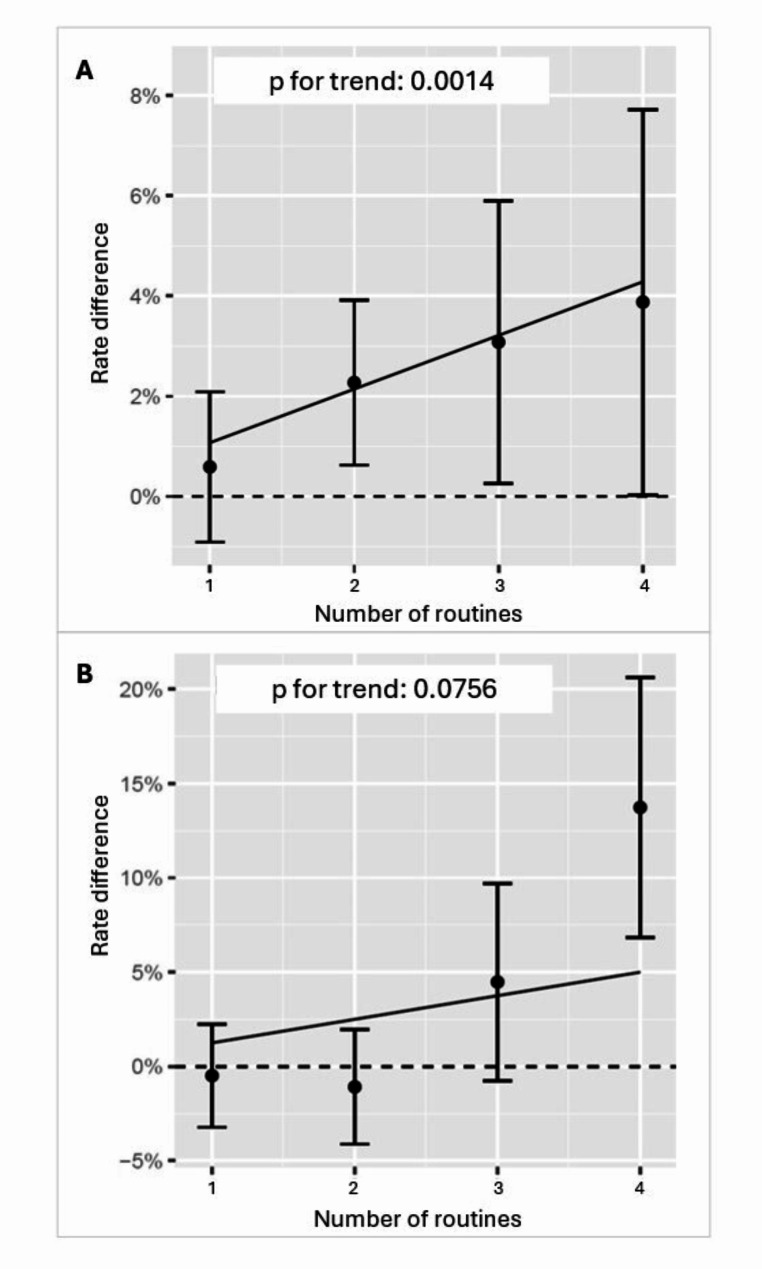
Adjusted differences and confidence intervals for the number of routines followed, from one to four routines, no routine followed being the reference group. Differences in proportion of patients with newly detected diabetes is shown above (**A**) and of patients receiving oral glucose-lowering treatment below (**B**)

Outcome data on newly detected diabetes cases was available for 7595 (99.9%) patients. During the first year post-MI 303 new cases of diabetes were detected. The proportion of patients with newly detected diabetes followed at centers where 0, 1, 2, 3 and 4 routines were implemented was 2.7%, 3.7%, 5.1%. 5.4% and 6.3%. Further details are shown in Table 4. Outcome data was available on 7594 (99.9%) patients for oral glucose-lowering treatment. Glucose-lowering medication other than insulin was prescribed to 1080 (14.2%) patients at discharge and 17.9% at one-year follow-up. Of these, nearly had a diabetes diagnosis (96.6%). The percentage of treated patients followed at centers where 0, 1, 2, 3 and 4 routines were implemented was 16.8%, 17.3%, 17.1%, 20.4% and 32.4%. The results from the mixed-effect modelling are shown in Table 5, displaying the adjusted differences in rates of new diagnoses and treatment and risk ratios with confidence intervals for the exposures dichotomized as 0–1 versus 2–4 routines and each routine separately. Having 2–4 routines in place was associated with a higher proportion of patients with newly diagnosed diabetes (adjusted difference 1.79%, risk ratio (RR) [95% confidence interval]: 1.54 [1.20, 1.88], *p* = 0.0017). The proportion of patients being treated with oral glucose-lowering medication was marginally higher for patients exposed to 2–4 routines compared to those exposed to 0–1 routines (adjusted difference 2.12%) without reaching statistical significance (RR 1.12 [0.98, 1.27], *p* = 0.0974). When examining each routine separately, routinely performing OGTT was associated with both outcomes, with an adjusted difference in detection of diabetes of 2.00% (RR 1.62 [1.26, 1.98], (*p* = 0.0007) and a higher probability of patients receiving diabetes treatment at one-year (adjusted difference 3.64%, RR 1.22 [1.07, 1.37], *p* = 0.0046). The proportion of patients receiving oral glucose-lowering treatment at one-year post-MI was also higher when such medication was reported to be adjusted by the CR physicians (adjusted difference 5.34%, RR 1.31 [1.06, 1.56], *p* = 0.0155). As shown in Fig. 1, compared to having no work routines for diabetes care in place, the more routines implemented the higher the rate of newly detected diabetes (p for trend = 0.0014).

**Table 4:** The proportion of patients with newly detected diabetes (left) and proportion of patients treated with oral glucose-lowering medication (right) as registered at follow-up visits within CR during the first year post-MI

	Newly detected diabetes cases		Glucose-lowering treatment	
	Exposed	Non-exposed	Exposed	Non-exposed
FG/HbA1c measured	4.5%	3.2%	18.0%	17.6%
OGTT routinely performed	5.3%	3.2%	19.8%	16.7%
Oral glucose-lowering medication adjusted	5.4%	3.8%	23.3%	17.1%
Joint case rounds	4.6%	3.9%	22.7%	17.3%

Exposed/non-exposed refers to patients followed at CR centers where the respective routine was applied/not applied. FG: fasting glucose; HbA1c: hemoglobin A1c; OGTT: oral glucose tolerance test

**Table 5:** Adjusted differences and relative risks (confidence intervals) in the proportion of newly detected diabetes and proportion receiving oral glucose-lowering treatment for patients followed at centers applying 0–1 vs. 2–4 routines, as well as for each routine separately

	Newly detected diabetes			Proportion receiving glucose-lowering medication		
	Adjusted difference	Relative risk	*p*-value	Adjusted difference	Relative risk	*p*-value
***Exposure definition***						
**0–1 vs. 2–4 routines**	1.79% (0.70%, 2.88%)	1.54 (1.20, 1.88)	0.0017	2.12% (-0.38%, 4.62%)	1.12 (0.98, 1.27)	0.0974
**Each routine separately**						
FG/HbA1c measured	0.71% (-0.6, 2.02%)	1.22 (0.81, 1.64)	0.2890	0.91% (-1.98%, 3.81%)	1.05 (0.89, 1.22)	0.5360
OGTT routinely performed	2.00% (0.88%, 3.11%)	1.62 (1.26, 1.98)	0.0007	3.64% (1.13%, 6.15%)	1.22 (1.07, 1.37)	0.0046
Oral glucose-lowering medication adjusted	1.12% (-0.92%, 3.17%)	1.30 (0.76, 1.84)	0.2830	5.34% (1.02%, 9.65%)	1.31 (1.06, 1.56)	0.0155
Joint case rounds	1.39% (-0.77%, 3.55%)	1.36 (0.80, 1.91)	0.2080	3.70% (-0.82%, 8.22%)	1.21 (0.95, 1.48)	0.1090

FG: fasting glucose; HbA1c: hemoglobin A1c; OGTT: oral glucose tolerance test

## Discussion

In this study we observed that having structured work routines for diabetes care in CR was associated with a higher number of newly detected diabetes cases and a higher proportion of patients being treated with oral glucose-lowering medication at one-year post-MI. Out of the four diabetes care routines examined, using OGTT during follow-up was most strongly associated with both outcomes, followed by glucose-lowering medication being initiated and/or adjusted by the CR physicians. Also, we observed a dose-response incremental benefit with a greater number of implemented routines.

Although CR programs are overall relatively comprehensive in Sweden, our study indicates considerable variation in how work routines for screening and treatment of diabetes in post-MI patients are applied across CR sites, with the majority of sites applying none or one of the routines surveyed, while others applied three or four routines. In the European Society of Cardiology guidelines for diabetes and cardiovascular disease and the American Diabetes Association update (both from 2023), screening for potential diabetes among patients who have suffered a cardiovascular event is highly recommended [5, 10]. It has previously been shown that by analyzing fasting glucose, HbA1c and/or performing OGTT, two-thirds of patients with acute coronary syndromes without known diabetes are found to have undiagnosed diabetes or other abnormalities in glucose metabolism [6]. This highlights the importance of early screening for glucometabolic abnormalities in patients with MI to enable adequate treatment, which in turn improves long-term prognosis reducing both cardiovascular disease- and diabetes complications. Only 63% of CR centers reported routinely evaluating fasting glucose and HbA1c at the start of the CR program, a percentage that should be closer to 100%. Also, only 40% reported routinely using OGTT, most of whom reported screening only a selection of patients. The results indicate ample room for improvement in screening for glucometabolic abnormalities within CR in Sweden.

Adequate diabetes treatment post-MI is strongly recommended [5]. Our findings showed that 80.8% of patients with diabetes were treated with glucose-lowering agents (oral or insulin) at discharge and 86.3% at one-year follow-up. Initiating or adjusting glucose-lowering treatment was, however, inconsistently carried out by physicians at CR centers, with only one in six centers reporting adherence to this routine. As indicated by the latest EUROASPIRE IV survey, conducted during the same time as the Perfect-CR study (2016–2017), there also seemed to be room for improvement in adequate diabetes treatment among patients with coronary artery disease in Europe. These authors showed that 75% of patients with established diabetes were treated with glucose-lowering medication, most commonly metformin [11]. The importance of identifying diabetes post-MI holds even more true with recent advancements of glucose-lowering treatment with cardioprotective capacity, [23–25]. Recent data from the SWEDEHEART registry has shown a marked increase in the use of SGLT-2 inhibitors and GLP-1RA in recent years, with more than 60% of post-MI patients being treated with either or both classes of drugs in 2023 [4]. Still there is room for improvement, combined these results indicate that CR physicians need to take a larger responsibility for adequate diabetes treatment post-MI. As such, this aspect of the risk factor treatment arsenal should to a larger extent be included in comprehensive CR care, analogous to treatment for hypertension and dyslipidemia.

The optimal laboratory assessments to detect glucometabolic abnormalities in patients with cardiovascular disease is still debated. Two of the diabetes care work routines examined in our study were evaluating fasting glucose and/or HbA1c values at the start of the CR program and routinely performing OGTT for detecting glucometabolic abnormalities during post-MI follow-up. Out of the two routines, performing OGTT was associated with an increased rate of newly diagnosed diabetes at one-year post-MI. Karayiannides et al. recently showed that performing OGTT identified 10% more patients with undetected diabetes at the time of MI, compared to using fasting glucose and HbA1c alone [7]. Similarly, EUROASPIRE V demonstrated that if OGTT was not conducted, 30% of patients diagnosed with type 2 diabetes and 70% of individuals with impaired glucose tolerance would have remained undetected [11]. Combined, these results support recommendations from the European Society of Cardiology and American Diabetes Association guidelines on complementing diabetes screening with fasting glucose and HbA1c with OGTT in unclear cases [5, 10]. OGTT is also necessary to diagnose patients with impaired glucose tolerance [26, 27] which is estimated to carry the same high risk for cardiovascular disease as newly detected diabetes [28].

Having joint case rounds with diabetologists was the work routine surveyed in the Perfect-CR study most seldom implemented at CR centers. The importance of the multidisciplinary team has been recognized to improve patient care, through which disease complications can be prevented [29, 30]. In a review by Muuza, the risk of amputation in patients with diabetes-related foot ulcers after initiation a multidisciplinary approach was investigated [29]. While the team composition varied, in 94% of the included studies major amputations were reduced. Also, patients under the care of multidisciplinary teams consistently demonstrated improved glycemic control, management of vascular and infection diseases, and a lower incidence of major amputations [29]. Chava et al. demonstrated the benefit of having a multidisciplinary team in caring for patients with congestive heart failure, showing a significant decrease in both readmission rates and length of hospital stay [30]. While falling short of statistical significance, we observed that at centers reporting to have joint case rounds with diabetologists for managing post-MI patients with diabetes, the chance of diagnosing more patients with diabetes and the proportion of patients being prescribed oral glucose-lowering medication was numerically higher. Combined, others´ and our results suggest that multidisciplinary team collaboration may contribute to better identification, treatment, and outcomes in several disease states.

### Strengths and limitations

Our study focused on the association of CR work routines for diabetes care at center-level and detection and treatment of diabetes on patient-level, an aspect that to our knowledge has not been previously studied. Data was used from the nationwide SWEDEHEART registry and the Perfect-CR cohort, both having a center-level coverage of 100%. Missing data at center-level was minimal, increasing representativity and minimizing bias. All the same, some limitations should be mentioned. Type of diabetes was not registered in SWEDEHEART at the time of the study. Classification of glucose-lowering medication was also limited to insulin and oral glucose-lowering agents. How diabetes is diagnosed is not registered in SWEDEHEART and can vary between centers. Data on fasting glucose and HbA1c was often missing and could thus not be included as an individual outcome of diabetes treatment quality. As the Perfect-CR study was not powered for long-term outcome analyses we did not investigate associations with MI or diabetes-related outcomes. Although OGTT offers essential insights for diagnosing diabetes and impaired glucose tolerance fort post-MI patients, it is costly and time-consuming, posing barriers to implementation. Finally, residual confounding cannot be excluded because this was an observational study and caution is required with respect to causal interpretation.

## Conclusions

This study provides supporting evidence for structured follow-up for identifying and managing patients with abnormal glucose metabolism post-MI. While our results particularly encourage the use of OGTT as a routine diagnostic work-up, a cluster-randomized trial is warranted to ascertain causality for individual and cumulative routine implementation.

## Supplementary material

Supplementary Material 1.

## Data Availability

Access to data from the SWEDEHEART registry needs to be applied for and third-party data usage is not allowed. Instead, given ethical study approval from the Swedish Ethical Review Authority, access to SWEDEHEART data supporting the present findings can be applied for from the Uppsala Clinical Research Center in Sweden. Further information can be found on the UCR www.ucr.uu.se/en/ and Swedish Ethical Review Authority etikprovningsmyndigheten.se/ websites. Aggregated data used in the current study are available from the corresponding author upon reasonable request.

## Abbreviations

CR: Cardiac rehabilitation
GLP: 1RA–Glucagon–like peptide–1 receptor agonist
HbA1c: Hemoglobin A1C
MI: Myocardial infarction
OGTT: Oral glucose tolerance test
SWEDEHEART: The Swedish Web system for Enhancement and Development of Evidence–based care in Heart Disease Evaluated According to Recommended Therapies
SGLT: 2–Sodium–glucose cotransporter–2
