# Supplementary material for: Structured diabetes care routines in cardiac rehabilitation are associated with increased diabetes detection and improved treatment after myocardial infarction: a nationwide observational study
Source: Cardiovasc Diabetol. 2024 Sep 3;23:330. doi: 10.1186/s12933-024-02425-6 (PMC11373097; doi:10.1186/s12933-024-02425-6)
Supplement: Supplementary file 1 — Supplementary Material 1. [file 12933_2024_2425_MOESM1_ESM.docx]

**Structured diabetes care routines in cardiac rehabilitation are associated with increased diabetes detection and improved treatment after myocardial infarction: a nationwide observational study**

Supplementary material

**Table S1.** Combinations of diabetes care work routines presented as numbers (proportion) of patients followed at CR centers applying each routine.

| **Routine** | **N (total)** | **FG/HbA1c measured** | **OGTT routinely performed** | **Joint case rounds** | **Glucose-lowering medication adjusted** |
| --- | --- | --- | --- | --- | --- |
| FG/HbA1c measured | | | | | |
| Control | 2986 | 0 % | 733 (25 %) | 204 (7 %) | 229 (8 %) |
| Exposed | 4609 | 100 % | 2104 (46 %) | 680 (15 %) | 710 (15 %) |
| OGTT routinely performed | | | | | |
| Control | 4758 | 2505 (53 %) | 0 % | 88 (2 %) | 485 (10 %) |
| Exposed | 2837 | 2104 (74 %) | 100 % | 796 (28 %) | 454 (16 %) |
| Joint case rounds | | | | | |
| Control | 6711 | 3929 (59 %) | 2041 (30 %) | 0 % | 655 (10 %) |
| Exposed | 844 | 680 (77 %) | 796 (90 %) | 100 % | 284 (32 %) |
| Glucose-lowering medication adjusted | | | | | |
| Control | 6656 | 3899 (59 %) | 2383 (36 %) | 600 (9 %) | 0 % |
| Exposed | 939 | 710 (76 %) | 454 (48 %) | 284 (30 %) | 100 % |

FG: fasting glucose; HbA1c: hemoglobin A1c; OGTT: oral glucose tolerance test.


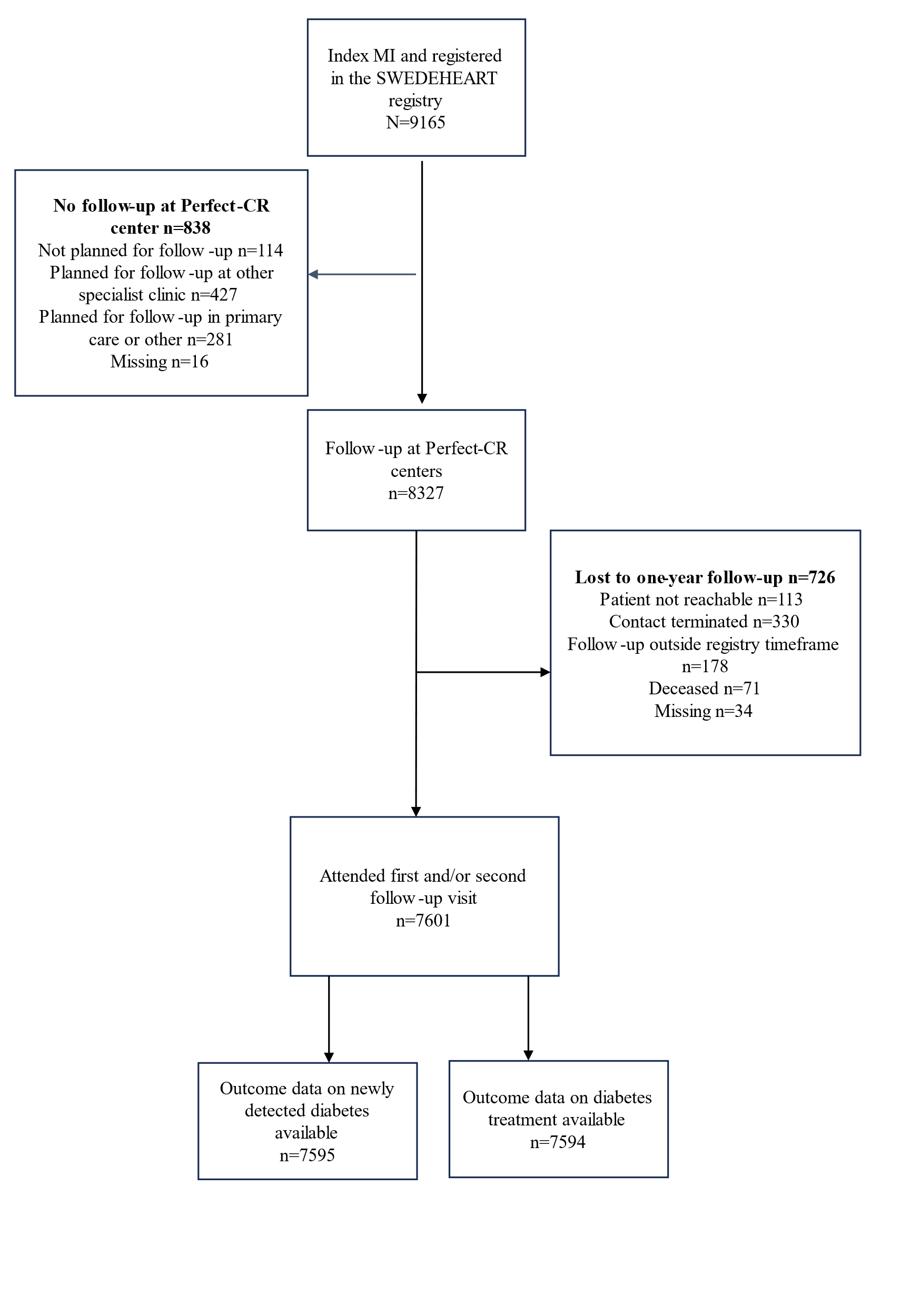


**Figure S1.** Flowchart of the study population.


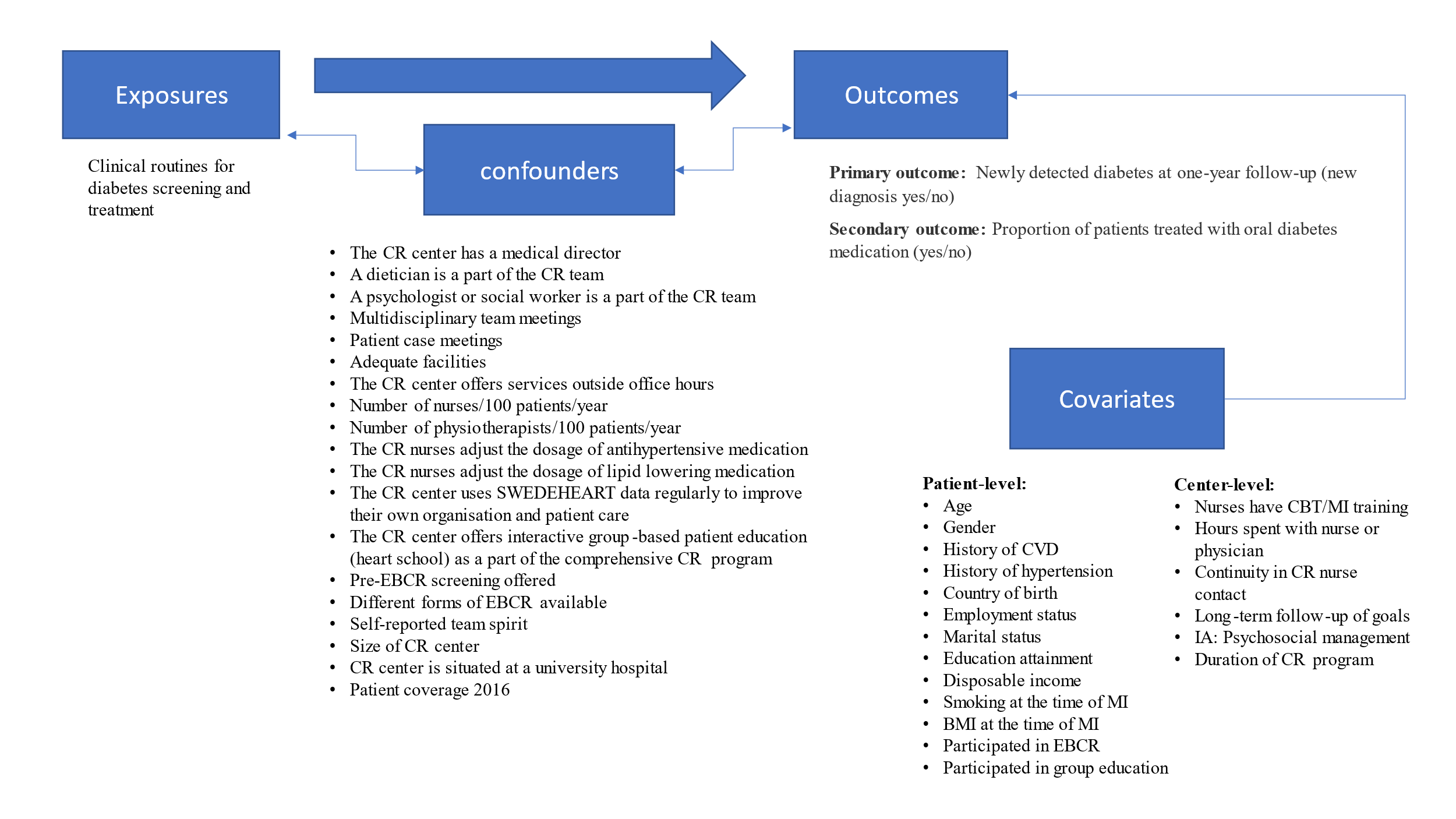


**Figure S2**. Directed acyclic graph showing assumed associations between exposures, confounders, covariates, and outcomes. All confounders as well as patient-level covariates were included in models estimating associations between exposures and outcomes. CR: Cardiac rehabilitation; EBCR: exercise-based cardiac rehabilitation; CVD: cardiovascular disease; MI: myocardial infarction; CBT: cognitive behavioral therapy IA: initial assessment.
